# Can the CROSS protocol be safely implemented in real world scenario with broader eligibility criteria? Experience from a tertiary care centre in India

**DOI:** 10.3332/ecancer.2021.1291

**Published:** 2021-09-16

**Authors:** Tapesh Bhattacharyya, Moses Arunsingh, Santam Chakraborty, Vishnu Harilal, Rohit Sasidharan, Saheli Saha, Robin Thambudorai, Bipradas Roy, Sudeep Banerjee, Paromita Roy, Soumendranath Ray, Indranil Mallick

**Affiliations:** 1 Department of Radiation Oncology, Tata Medical Center, 14, MAR(E-W), DH Block (Newtown), Action Area I, Newtown, Kolkata, West Bengal 700160, India; 2 Department of Gastrointestinal Surgery, Tata Medical Center, 14, MAR(E-W), DH Block (Newtown), Action Area I, Newtown, Kolkata, West Bengal 700160, India; 3 Department of Pathology, Tata Medical Center, 14, MAR(E-W), DH Block (Newtown), Action Area I, Newtown, Kolkata, West Bengal 700160, India; 4 Department of Nuclear Medicine, Tata Medical Center, 14, MAR(E-W), DH Block (Newtown), Action Area I, Newtown, Kolkata, West Bengal 700160, India

**Keywords:** neoadjuvant chemoradiation, CROSS protocol, carcinoma oesophagus, real world scenario, broader eligibility

## Abstract

**Background:**

The Chemoradiotherapy for Oesophageal Cancer Followed by Surgery Study (CROSS) trial established a new benchmark in the management of oesophageal cancer with neoadjuvant chemoradiation followed by surgery with a marked benefit for squamous cell carcinomas (SCCs). We evaluate if the CROSS protocol can be safely implemented with a broader eligibility criteria in a real-world setting.

**Methods:**

A retrospective analysis of 80 patients of SCC oesophagus was performed, who were treated with neoadjuvant chemoradiation with radiation therapy (RT) to 41.4 Gy/23 Fr/4.5 weeks and weekly paclitaxel and carboplatin, followed by surgery at our institute between 2012 and 2019. Eligibility for the use of this regimen was expanded beyond the limits of size and stage allowed in the CROSS trial.

**Results:**

The median age of this cohort was 57 years (range: 39–78 years). Most of the patients (77/80; 96.3%) had T3 disease and 25% patients (20/80) had N2/N3 disease. Thirty-three patients (41.3%) had the disease beyond CROSS eligibility criteria. All patients completed planned course of RT and five cycles of weekly chemotherapy were received by 61 patients (76.2%). Overall pathological complete response (pCR) could be achieved in 33 patients (41.3%). Among 33 CROSS ineligible patients, 14 (42.4%) had pCR. Acute grade 3 dysphagia and grade ≥ 3 neutropenia were seen in seven cases (8.3%) and nine cases (10.7%), respectively. At a median follow-up of 16 months, 1-year and 2-year overall survival (OS) were 84.4% (95% confidence interval (CI): 73.5%–91.1%) and 76.3% (95% CI: 63.2%–85.2%), respectively, for the entire cohort. For CROSS ineligible patients, 1-year and 2-year OS were 82% (95% CI: 61.8%–92.2%) and 72.7% (95% CI: 50.4%–86.2%), respectively. On univariate analysis, patients who had pathologically N0 disease had significantly better 2-year OS (85.7% versus 48.4%; p = 0.03) as compared to pathologically N+ patients. On univariate and multivariate analysis, there was no significant difference in OS and progression free survival between CROSS eligible and CROSS ineligible patients.

**Conclusion:**

CROSS protocol can be safely implemented for carefully selected patients of SCC oesophagus outside clinical trial settings with expanded eligibility criteria.

## Introduction

Curative intent resection in non-metastatic oesophageal cancers is associated with 5-year survival between 20% and 35% [1–3]. Given these poor outcomes, combinations of chemotherapy and radiotherapy (RT) have been evaluated in the neoadjuvant and adjuvant setting. Theoretical advantages of a neoadjuvant approach include downstaging of disease, reduced hypoxia and earlier treatment of potential systemic micro-metastases.

Neoadjuvant chemotherapy (NACT) and neoadjuvant concurrent chemoradiation (NACTRT) have not been directly compared in this setting. However, a meta-analysis by Gebski *et al* [4] demonstrated a survival benefit with NACTRT, but not with NACT, in patients with squamous cell carcinomas (SCCs). Neoadjuvant chemoradiotherapy has the advantages of combining effective local and systemic therapy, superior R-0 resection rates and a higher pathological complete response (pCR) rate compared with NACT alone. The initial results of Chemoradiotherapy for Oesophageal Cancer Followed by Surgery Study (CROSS) trial [5] demonstrated that, NACTRT resulted in an improved median overall survival (OS) of 49.4 months compared with 24.0 months with surgery alone without increasing the risks of postoperative complications after NACTRT.

The improvement was even marked for the subset with squamous carcinoma with a median survival of 81.6 months in NACTRT plus surgery group versus 21.1 months in surgery alone arm as reported in subsequent results [6]. Since the report of these extremely encouraging results, this protocol has become a standard of care worldwide. However, there has been a paucity of reports validating these results outside the trial setting.

Since 2012, we have used the CROSS protocol routinely for patients with oesophageal cancers in our institute. We have used this protocol also for technically operable patients beyond the eligibility limits defined by the CROSS trial. The objectives of the current audit was to determine if the results of the CROSS trial were reproducible in the real-world setting, and verify if the protocol could be safely and effectively used with broader inclusion criteria.

## Materials and methods

Patients with SCC oesophagus treated with CROSS protocol between 2012 and 2019 at Tata Medical Center, Kolkata, were retrospectively analysed. Information was captured from the electronic database of the hospital management system on Research electronic data capture [7]. Patients with cT1–T4 and N0–N3 histologically confirmed SCC of the oesophagus who had received trimodality treatment at our institution were considered.

Baseline evaluation included upper gastro-intestinal endoscopy, radiological staging with a contrast enhanced computed tomography (CECT) thorax and abdomen or positron emission tomography-computed tomography (PET-CT), complete haemogram, renal function tests and liver function tests, along with echocardiography and pulmonary function tests (PFTs). Endoscopy and biopsy were performed to know the mucosal extent of the disease and histological confirmation of diagnosis. PET-CT was routinely performed in all the cases for staging work up since 2015. Echocardiography and PFTs were done before starting treatment to know the baseline cardiac and lung function status. In patients in whom PFT was not possible, a 6-minute walk test was done to assess the baseline lung function. All patients provided written informed consent before starting treatment. Tumour Node Metastasis (TNM) staging was performed using American Joint Committee on Cancer 7th edition [8]. Toxicities were recorded using Common Terminology Criteria for Adverse Events (CTCAE) version 4 [9]. Following initial clinical staging, all patients were discussed at a multidisciplinary tumour board with treatment recommendations for NACTRT followed by oesophagectomy approved by physicians within the specialities of surgical oncology, thoracic surgery, gastroenterology, radiation oncology, medical oncology, radiology and pathology.

The NACTRT schedule was as follows: on days 1, 8, 15, 22 and 29, carboplatin targeted at an Area Under Curve of 2 mg/mL/minute and paclitaxel at a dose of 50 mg/m^2^ of body surface area were administered intravenously. A total external beam RT dose of 41.4 Gray was given in 23 fractions (conven tional fractionation), starting on the first day of the first chemotherapy cycle. During NACTRT, laboratory tests (including complete blood cell counts and renal function tests) were done on a weekly basis. The patients were closely monitored for toxic effects of NACTRT.

### Radiotherapy

Patients were positioned supine, immobilised on an all in one board (AIO board, Orfit Industries) using a T-bar with arms above the head and arm cushions placed below the arm for support. Contrast enhanced helical planning CT images were acquired with a slice thickness of 2.5 mm. Gross Tumour Volume (GTV) was delineated on the planning CT after taking into account the information provided by the PET CT and endoscopy. The GTV was defined as gross primary tumour and the enlarged lymph nodes. A cranio-caudal margin of 3 cm and a radial margin of 1 cm were added around the GTV to create the clinical target volume (CTV). The CTV was trimmed from the anatomical barriers such as heart, lung, major vessels and bones as required. The final planning target volume (PTV) was then created by expanding the CTV by adding 1 cm cranio-caudally and 0.7 cm radially to the CTV. Elective mediastinal nodal irradiation was not performed.

A total RT dose of 41.4 Gy in 23 fractions was delivered over four and half weeks on linear accelerators. 3D conformal RT (3DCRT) or intensity modulated RT (IMRT) was used to achieve adequate target coverage and adhere to the dose constraints for the organs at risk (OAR). For 3DCRT, 15 MV beams were preferred, while IMRT was delivered using 6 MV beams. The OAR constraints used were spinal cord D max < 45 Gy, lung V20 ≤ 30%, V10 ≤ 50%, V5 ≤ 70%, mean lung dose ≤ 15 Gy and mean heart dose ≤ 26 Gy.

Following NACTRT, a repeat staging CECT thorax and abdomen was routinely obtained at 4–5 weeks to assess for treatment response and to ensure no metastatic disease was identifiable prior to oesophagectomy. The patients were subsequently taken up for a planned definitive surgery, about 6 to 8 weeks following the completion of NACTRT. The most common surgical approaches to accomplish the resection of oesophageal cancer included McKeown and Ivor Lewis operation.

Tumour response was graded using the College of American Pathologist Cancer Protocol/Modified Ryan Scheme for Tumour Regression Score. Tumour regression grade (TRG) 0 means no viable cancer cells (complete response). TRG 1 means single cells or rare small groups of cancer cells (near complete response). TRG2 means residual cancer with evident tumour regression, but more than single cells or rare small groups of cancer cells (partial response). TRG 3 means extensive residual cancer with no evident tumour regression (poor or no response).

One hundred and fourteen patients were actually planned for CROSS protocol; however, only 84 patients could undergo planned neoadjuvant chemoradiation followed by surgery at our institute.

### Statistical analysis

Time to event endpoints like survival was analysed using Kaplan–Meier method with the log rank test to ascertain any significance. OS was calculated from the starting date of RT to the date of death or last follow-up. Progression free survival (PFS) was calculated from the date of surgery to the date of progression or death or date of last follow-up. Local control was defined as absence of radiological or endoscopic progression. Univariate and multivariate analysis were performed to evaluate impact of different variables on patients treatment outcomes and survival. Hazard ratios (HRs) with 95% confidence limits (confidence interval (CI)) were calculated using the Cox proportional hazard model. On univariate analysis, factors which achieved *p* value of around 0.1 were considered for multivariate analysis. A *p* value of less than 0.05 was considered as statistically significant. Statistical analysis was performed using freely available EZR software (Saitama Medical Center, Jichi Medical University, Saitama, Japan) developed by Kanda [10] which is a graphical user interface for R (The R foundation for statistical computing, Vienna, Austria).

## Results

**Figure 1.**
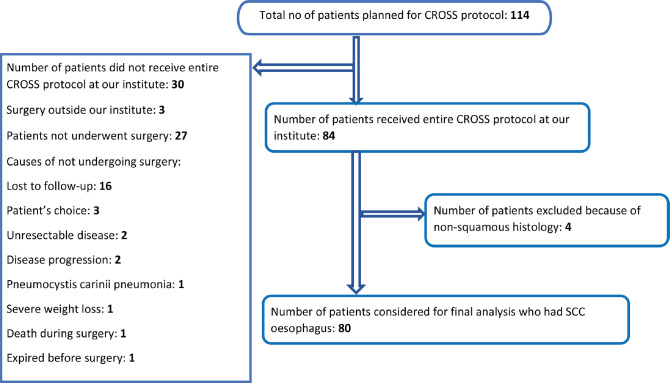
Flowchart showing the patients included in the study.

A total of 114 patients were planned for neoadjuvant chemoradiation followed by surgery according to CROSS protocol. However, only 84 patients could receive the scheduled treatment at our institute. Twenty-seven patients did not undergo surgery according to records captured from our electronic database. Three patients underwent surgery outside our institute, whose surgical and pathological data are not available with us. Out of those 27 patients, 16 patients (59.3%) did not comply with their planned surgical schedule and were lost to follow-up after completion of neoadjuvant chemoradiation. The reasons behind not undergoing surgery are displayed in Figure 1. Treatment related toxicity was not a common reason for declining surgery. All 80 patients of SCC oesophagus who did complete the planned protocol at our institute were considered for this retrospective analysis.

### Demographic characteristics

The median age of this cohort was 57 years (range: 39–75 years) with a male to female ratio of 1.6:1. All patients (100%) had SCC histology. The majority of the patients (50%) had tumour epicentre located in the middle thoracic oesophagus followed by lower thoracic oesophageal disease in 47.5% patients. A majority of the patients (77/80; 96.3%) had T3 disease. One fourth of this cohort had high lymph nodal burden (N2/N3 disease). The median radiological length of the tumour was 5.5 cm (ranging from 1.7 to 13.1 cm). A total of 33 patients (41.3%) could not match the CROSS eligibility criteria. Sixteen patients (20%) had tumours more than 8 cm in length. Among them, 11 patients (13.75%) were not eligible because of only tumour length > 8 cm. The most common cause of ineligibility was heavy nodal burden (20/80; 25%). Two patients presented with T4a disease. The baseline characteristics of this cohort are shown in Table 1.

### Treatment

All of the 80 patients completed the full course of external beam RT with 41.4 Gy/23 fractions. Fifty-nine patients (73.8%) were treated with 3DCRT and the rest with IMRT either in the form of rapidarc or tomotherapy. Sixty-one patients (76.25%) could complete all five cycles of chemotherapy. Rest of the patients (19; 23.75%) could not complete all the five cycles of chemotherapy because of grade 3 or more severe leucopenia, thrombocytopenia, dysphagia, fever or generalised weakness. Chemotherapy dose reduction of 25% or more was required in only two patients. The majority of the patients (56/80; 70%) underwent McKeowns oesophagectomy followed by Ivor Lewis operation in 15 patients (18.8%). The median length of gap between end of neoadjuvant chemoradiation and surgery was 48 days ranging from 34 to 81 days.

### Pathology

R0 resection could be performed in 74 patients (92.5%). The median number of lymph nodes dissected was 21 (range: 4–58). The median number of positive lymph nodes was 0 (range: 0–17). Instead of the Mandard score which was followed in the original CROSS protocol, we followed the College of American Pathologists protocol for reporting of response to treatment. According to this system, TRG 0 was found in 40 cases (50%). pCR (pT0N0) could be achieved in 33 patients (41.25%). Nodal regression status is not included in tumour regression grading. Hence, out of those 40 TRG 0 cases, 5 had pN1 and 2 had pN2 disease.

### Survival analysis

**Figure 2.**
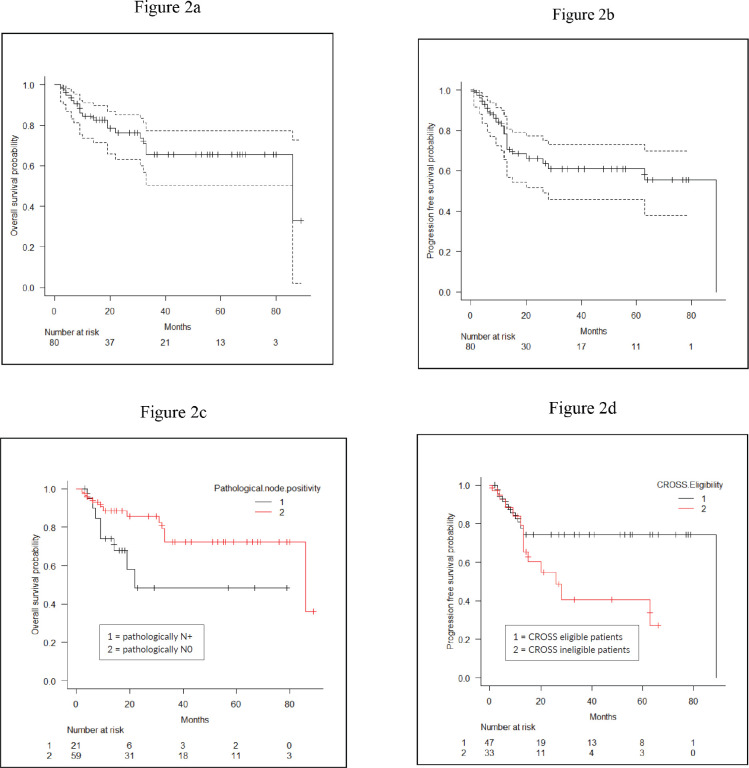
(a): OS of the entire cohort. (b): PFS of the entire cohort. (c): Comparison of OS between pathologically node negative and node positive patients. (d): Comparison of PFS between CROSS eligible and CROSS ineligible patients.

At a median follow-up of 16 months, 1-year and 2-year OS were 84.4% (95% CI: 73.5%–91.1%) and 76.3% (95% CI: 63.2%–84.2%), respectively, for the entire cohort (Figure 2a). For CROSS ineligible patients, 1-year and 2-year OS were 82% (95% CI: 61.8%–92.2%) and 72.7% (95% CI: 50.4%–86.2%), respectively. The 1-year and 2-year PFS of the entire cohort were 78.4% (95% CI: 65.5%–86.9%) and 66.1% (95% CI: 51.8%–77.1%) (Figure 2b). On univariate analysis, patients who had pathologically node negative disease had significantly better 2-year OS (85.7% versus 48.4%; HR: 1.13 (95% CI: 1.01–1.25) *p* = 0.03) as compared to pathologically node positive patients (Figure 2c). On univariate analysis, CROSS eligible patients (*n* = 33) had shown a trend towards improved 2-year PFS (74.3% versus 54.8%; HR: 2.29 (95% CI: 0.98–5.39) *p* = 0.05) as compared to patients who did not match the CROSS eligibility criteria; however, it could not reach any statistical significance (Figure 2d). On multivariate analysis using Cox regression model, not a single variable stood out to be significant prognostic factor for both OS and PFS. The detailed univariate and multivariate analysis of OS and PFS are shown in Tables 2 and 3.

### Patterns of failure

At the time of analysis, 23 patients (28.8%) had disease progression. Only four patients experienced local failure (5%) overall and only one patient developed local failure alone among 33 CROSS ineligible patients. Distant metastasis was the most common pattern of failure (13 cases; 16.3%). Bone and lung were the most common sites of distant metastasis.

### Dosimetry

Out of 80 patients, 14 patients (17.5%) could not meet the lung dose constraints as per Quantitative Analysis of Normal Tissue Effects in Clinic guidelines. V5 constraint alone, V10 constraint alone and V20 constraint alone were not achieved in five patients (6.25%), two patients (2.5%) and one patient (1.25%), respectively. Both V20 and V10 constraints were not met in two patients (2.5%). Both V10 and V5 constraints were not met in four patients (5%). Out of those 14 patients, the PTV length was above the mean length of 15.94 cm in 12 patients. PTV volume was above the mean volume of 441 cc in ten patients. Bulky disease (T4 or N2) was noted in five patients.

### Treatment related complications

#### NACTRT toxicities

Toxicities were graded according to CTCAE version 4 [9]. Grade 3 dysphagia was seen in six cases (7.5%). A total of grade 3 or more severe neutropenia were seen in nine (11.3%) cases. Grade 3 thrombocytopenia and febrile neutropenia were experienced by 3.8% and 5% of the patients, respectively.

#### Surgical complications

All postoperative complications including pulmonary, cardiac and anastomotic leakage complications were recorded up to 30 days postoperatively or during the same hospital stay after surgery. The median length of postoperative hospital stay was 12 days ranging from 1 to 42 days. Less than 30 days postoperative mortality was seen in four patients (5%). Acute toxicities of neoadjuvant chemoradiation and post-surgical complications are shown in Table 4.

## Discussion

Neoadjuvant chemoradiation followed by surgery is currently considered a widely practiced approach in resectable oesophageal cancer. Encouraged by the results of the CROSS trial, we started implementing this neoadjuvant chemoradiation strategy in our institute from 2012. Our study showed that weekly Carboplatin and Paclitaxel along with RT were well tolerated and patient compliant. All our patients could complete RT within stipulated period of time without any dose reduction. Three fourths of our patients could complete all five cycles of chemotherapy planned and 25% dose reduction was required only in two cases. Grade III or more severe form of neutropenia was seen in 11.3% of cases slightly higher than the CROSS study where 7% of cases suffered from grade III haematological toxicities. Meerten *et al* [11] reported grade III or more severe neutropenia in 15.1% and grade III–IV leukopenia in 23.5% of patients with the same regime in their phase II study. The major non-haematological toxicity was grade III dysphagia seen in 7.5% of cases of our cohort as compared to 7.3% grade III oesophagitis reported by Meerten *et al* [11]. Shapiro *et al* [6] in their long-term results of CROSS study reported grade 3 or worse non-haematological toxicities in 11% of cases. Less than 30 day postoperative mortality was seen in 4.8% of our patients as compared to 2% of the chemoradiation followed by surgery arm in the CROSS trial [5]. Head-on comparison of surgical complications between our study and the neoadjuvant chemoradiation arm of CROSS study is difficult as we observed many overlapping toxicities.

In this cohort, 1-year and 2-year OS were 84.4% and 76.3%, respectively, compared to 82% and 67% in the neoadjuvant chemoradiation arm of CROSS study. The interesting point in this study is that 41.3% of the patients did not match the CROSS eligibility criteria. Tumour length more than 8 cm and heavy nodal burden (N2/N3) were found in 20% and 25% patients of this cohort, respectively. There was no significant difference in OS or PFS between CROSS eligible and ineligible patients on univariate and multivariate analysis. Moreover, when we have analysed individual factors, i.e., heavy versus less nodal burden or length of the disease and its impact on outcome, none of them came out to be significant It suggests that CROSS protocol is not only applicable in trial settings, it is also a reasonable option for carefully selected patients with more advanced disease in real life scenario which is commonly found in low or middle income countries with limited resources and where screening programmes are not well developed or strictly implemented.

The R0 resection rate in this study was 92.5% which is similar to the neoadjuvant chemoradiation arm of CROSS protocol. The pCR in both the primary and the regional lymph node (ypT0N0) in this study was 41.3% compared to 49% found in the SCC group of the CROSS cohort. The substantial downstaging as a result of chemoradiotherapy is also reflected in the significantly higher percentage of R0 resections in the chemoradiotherapy–surgery group. The slightly lower pCR rate may be explained by the broader eligibility and more advanced disease in our cohort. Lee *et al* [12] in preoperative chemotherapy with hyperfractionated RT arm showed a pCR rate of 43% comparable to our studies. Meerten *et al* [11] showed a pCR rate of 25% in their entire cohort, pCR rate in SCC patients was not separately mentioned in the results. The role of pCR in the OS and disease free survival (DFS) has been highlighted time and again. Berger *et al* [13] have documented a survival benefit in patients who have been downstaged to pathological stage 0 or 1. They also mentioned the completeness of resection to be of more importance in determination of survival. Meredith *et al* [14] have noted a significant 5-year OS and DFS benefit in complete responders in comparison to partial or no-responders. More of these patients had had R0 resections and less local recurrence. In our study, it was the pathological node negative status that impacted on improved OS on univariate analysis; however, on multivariate analysis not a single variable stood out as a significant independent prognostic factor. Reynolds *et al* [15] demonstrated that ypN status is the strongest determinant of outcome, more important than the achievement of a complete pathologic response or of histomorphologic regression at the primary site. Gu *et al* [16] showed that the number of LNs with metastasis is an independent prognostic factor in patients with residual adenocarcinoma of the oesophagus or the EGJ after preoperative chemoradiation.

The drawback of this study is its retrospective nature and small sample size. Toxicity documentation could have been better if it is a prospective one. The duration of follow-up of these patients has been short owing to the fact that most of these patients were residents of other states or countries. Radiological and endoscopic examination on follow-up was based on symptoms rather than regular surveillance. In the CROSS trial, most of the locoregional recurrences and distant metastasis occurred within 24 months and 30 months, respectively. With a median follow-up of only 16 months in our study, these recurrences are likely to be missed.

## Conclusion

Our results suggest that neoadjuvant chemoradiation following CROSS protocol can be safely implemented in carefully selected patients of SCC oesophagus in real world scenarios outside the clinical trial setting with broader eligibility criteria.

## Funding support

Nil.

## Conflicts of interest

Nil.

## Figures and Tables

**Table 1.** Baseline characteristics of the entire cohort.

Features		Number (%) (*n* = 80) (100%)
Age median (range)		57 years (39–78 years)
Sex	Male	49 (61.3)
	Female	31 (38.7)
Performance status	0	18 (22.5)
	1	55 (68.8)
	2	7 (8.8)
Histology	Squamous cell carcinoma	80 (100)
	Adenocarcinoma	0 (0)
Location	Lower thoracic	38 (47.5)
	Middle thoracic	40 (50)
	Upper thoracic	2 (2.5)
Tumour length median (range)		5.5 cm (1.7–13.1 cm)
Clinical T stage	cT1b	1 (1.3)
	cT3	77 (96.3)
	cT4a	2 (2.5)
Clinical N stage	cN0	29 (36.3)
	cN1	31 (38.8)
	cN2	19 (23.8)
	cN3	1 (1.3)
Technique of RT	3DCRT	59 (73.8)
	Rapid arc	7 (8.8)
	Tomotherapy	12 (15)
	Missing	2 (2.4)
CROSS eligible	Yes	47 (58.7)
	No	33 (41.3)

**Table 2.** Comparison of PFS and OS among different prognostic variables.

Prognostic variables	Features		2-year PFS (95% CI)	*p* value	2-year OS (95% CI)	*p* value
Age	≤57 years	40	66.0 (43.9–81.1)	0.55	74.6 (54.7–86.7)	0.56
	>57 years	40	66.5 (46.3–80.6)		77.9 (58.4–89.0)	
Sex	Male	49	64.7 (45.5–78.5)	0.80	81.5 (64.4–90.9)	0.46
	Female	31	68.3 (44.4–83.6)		68.3 (45.6–83.2)	
Location	Lower	38	60.3 (38.1–76.7)	0.35	73.8 (53.4–86.4)	0.21
	Middle & Upper	42	70.7 (50.8–83.8)		78.8 (60.0–89.4)	
Clinical N status	N0 N1	60	70.9 (54.8–82.2)	0.29	82.1 (68.0–90.4)	0.11
	N2 N3	20	46.9 (17.6–71.8)		53.0 (20.7–77.4)	
Radiological tumour length	≤5.5 cm	41	66.7 (47.7–80.1)	0.35	78.8 (58.0–90.1)	0.24
	>5.5 cm	39	67.1 (43.8–82.5)		72.4 (53.3–84.7)	
PTV length	≤15.6 cm	40	61.5 (40.8–76.8)	0.70	74.0 (53.6–86.4)	0.94
	>15.6 cm	40	72.1 (49.7–85.8)		76.6 (56.4–88.4)	
PTV volume	≤424.5 cc	41	68.8 (49.3–82.1)	0.94	81.5 (62.2–91.5)	0.26
	>424.5 cc	39	63.0 (39.3–79.6)		68.5 (47.6–82.5)	
CROSS eligible	Yes	47	74.3 (56.0–85.9)	0.05	78.6 (60.9–89.0)	0.14
	No	33	54.8 (31.7–72.9)		72.7 (50.4–86.2)	
TRG class	0, 1	47	73.1 (52.9–85.7)	0.09	76.1 (57.2–87.5)	0.45
	2, 3	33	58.2 (36.4–74.8)		76.5 (56.2–88.3)	
ypN status	Node negative	59	70.6 (53.7–82.3)	0.09	85.7(72.0-93.0)	**0.03**
	Node positive	21	54.0 (26.4–75.3)		48.4 (20.6–71.7)	
pCR	Yes	33	77.0 (53.1–89.8)	0.06	82.0 (61.3–92.3)	0.21
	No	47	58.2 (39.2–73.2)		72.1 (53.6–84.2)	

PFS, Progression free survival; OS, Overall survival; CI, Confidence interval; HR, Hazard ratio; PTV, Planning target volume; pCR, Pathological complete response; TRG, Tumour regression grade; NA, Not applicable

**Table 3.** HRs and CIs in univariate and multivariate analysis of PFS and OS.

Characteristics	2-year PFS univariate analysis HR	95% CI	*p* value	2-year PFS multivariate analysis HR (95% CI) p value	2-year OS univariate analysis HR	95% CI	*p* value	2-year OS multivariate analysis HR (95% CI) p value
Age	1.29	0.55–3.03	0.55	NA	0.77	0.31–1.89	0.57	NA
Sex	0.89	0.37–2.15	0.81	NA	1.39	0.57–3.44	0.47	NA
Location	0.67	0.29–1.56	0.36	NA	0.56	0.23–1.41	0.22	NA
Clinical N status	1.64	0.64–4.21	0.30	NA	2.17	0.82–5.80	0.12	NA
Radiological tumour length	1.16	0.50–2.69	0.73	NA	1.72	0.69–4.28	0.24	NA
PTV length	0.85	0.36–2.02	0.71	NA	1.04	0.42–2.55	0.94	NA
PTV volume	0.97	0.41–2.30	0.94	NA	1.67	0.67–4.15	0.27	NA
CROSS eligibility	2.29	0.98–5.39	0.05	2.36 (0.90–6.21) *p* = 0.08	1.91	0.79–4.62	0.15	2.05 (0.79–5.30) *p* = 0.14
TRG class	2.05	0.87–4.84	0.10	0.95 (0.18–4.90) *p* = 0.95	1.40	0.58–3.40	0.45	1.04 (0.22–4.95) *p* = 0.97
ypN	1.10	0.98–1.24	0.11	0.67 (0.21–2.13) *p* = 0.50	1.13	1.01–1.25	**0.03**	0.38 (0.11–1.35) *p* = 0.13
pCR	2.41	0.94–6.18	0.06	2.09 (0.30–14.34) *p* = 0.45	1.83	0.70–4.78	0.22	1.20 (0.17–8.34) *p* = 0.85

PFS, Progression free survival; OS, Overall survival; CI, Confidence Interval; HR, Hazard ratio; PTV, Planning target volume; pCR, Pathological complete response; TRG, Tumour regression grade; ypN, Pathological nodal status; NA, Not applicable

**Table 4.** Treatment related complications (acute toxicities of neoadjuvant chemoradiation and surgical complications).

Acute toxicities of neoadjuvant chemoradiation		Number (%)
≥Grade III RT induced dysphagia	Yes	6 (7.5)
	No	72 (90)
	Missing	2 (2.5)
≥Grade III RT induced nausea and vomiting	Yes	3 (3.8)
	No	75 (93.8)
	Missing	2 (2.5)
Grade III anaemia	Yes	2 (2.5)
	No	72 (90.0)
	Missing	6 (7.5)
≥Grade III neutropenia	Grade 3	6 (7.5)
	Grade 4	3 (3.8)
	No	69 (86.3)
	Missing	2 (2.5)
≥Grade III thrombocytopenia	Grade 3	2 (2.5)
	Grade 4	1 (1.2)
	No	73 (91.3)
	Missing	4 (5)
Surgical complications		
Pulmonary complications	Pneumonia	12 (15)
	Infection and sepsis	8 (10)
	Pleural effusion	7 (8.8)
	Respiratory failure	3 (3.8)
	Aspiration	4 (5)
	Pneumothorax and pneumomediastinum	2 (2.5)
Cardiac complications	Atrial fibrillation	3 (3.8)
	Supraventricular tachycardia	2 (2.5)
	Shock with DIC	2 (2.5)

RT, Radiotherapy; DIC, Disseminated intravascular coagulation

^a^For surgical complications adding up the data will not result in 100%

## References

[1] Bray F, Ferlay J, Soerjomataram I (2018). Global cancer statistics 2018: GLOBOCAN estimates of incidence and mortality worldwide for 36 cancers in 185 countries. CA Cancer J Clin.

[2] Allum WH, Stenning SP, Bancewicz J (2009). Long-term results of a randomized trial of surgery with or without preoperative chemotherapy in esophageal cancer. J Clin Oncol.

[3] Kelsen DP, Winter KA, Gunderson LL (2007). Long-term results of RTOG trial 8911 (USA Intergroup113): a random assignment trial comparison of chemotherapy followed by surgery compared with surgery alone for esophageal cancer. J Clin Oncol.

[4] Gebski V, Burmeister B, Smithers BM (2007). Survival benefits from neoadjuvant chemoradiotherapy or chemotherapy in oesophageal carcinoma: a meta-analysis. Lancet Oncol.

[5] van Hagen P, Hulshof MC, van Lanschot JJ (2012). Preoperative chemoradiotherapy for esophageal or junctional cancer. N Engl J Med.

[6] Shapiro J, van Lanschot JB, Hulshof Marten CCM (2015). Neoadjuvant chemoradiotherapy plus surgery versus surgery alone for oesophageal or junctional cancer (CROSS): long-term results of a randomized controlled trial. Lancet Oncol.

[7] Harris PA, Taylor R, Thielke R (2009). Research electronic data capture (REDCap)—a metadata-driven methodology and workflow process for providing translational research informatics support. J Biomed Inf.

[8] (2010). AJCC Cancer Staging Manual.

[9] National Cancer Institute (2010). Common Terminology Criteria for Adverse Events (CTCAE) Version 4.0.

[10] Kanda Y (2013). Investigation of the freely available easy-to-use software ‘EZR’ for medical statistics. Bone Marrow Transplant.

[11] van Meerten E, Muller K, Tilanus HW (2006). Neoadjuvant concurrent chemoradiation with weekly paclitaxel and carboplatin for patients with oesophageal cancer: a phase II study. Br J Cancer.

[12] Lee JL, Park SI, Kim SB (2004). A single institutional phase III trial of preoperative chemotherapy with hyperfractionation radiotherapy plus surgery versus surgery alone for respectable esophageal squamous cell carcinoma. Ann Oncol.

[13] Berger AC, Farma J, Scott WJ (2005). Complete response to neoadjuvant chemoradiotherapy in esophageal carcinoma is associated with significantly improved survival. J Clin Oncol.

[14] Meredith KL, Weber JM, Turaga KK (2010). Pathologic response after neoadjuvant therapy is the major determinant of survival in patients with esophageal cancer. Ann Surg Oncol.

[15] Reynolds JV, Muldoon C, Hollywood D (2007). Long-term outcomes following neoadjuvant chemoradiotherapy for esophageal cancer. Ann Surg.

[16] Gu Y, Swisher SG, Ajani JA (2006). The number of lymph nodes with metastasis predicts survival in patients with esophageal or esophagogastric junction adenocarcinoma who receive preoperative chemoradiation. Cancer.

